# Prevalence and Correlates of Self-Reported ADHD Symptoms in Bariatric Patients: Focus on Mood and Anxiety Comorbidity, Disordered Eating, and Temperamental Traits

**DOI:** 10.1007/s11695-024-07308-z

**Published:** 2024-07-25

**Authors:** Giulio Emilio Brancati, Viarda Cosentino, Margherita Barbuti, Francesco Weiss, Alba Calderone, Paola Fierabracci, Guido Salvetti, Ferruccio Santini, Giulio Perugi

**Affiliations:** 1 Department of Clinical and Experimental Medicine, University of Pisa, Via Savi 10, 56126 Pisa, Italy; 2 Endocrinology Unit, Department of Clinical and Experimental Medicine, Obesity and Lipodystrophy Research Center, University Hospital of Pisa, Via Paradisa 2, 56124 Pisa, Italy

**Keywords:** Attention-deficit/hyperactivity disorder, ADHD, Obesity, Bariatric surgery, Temperament

## Abstract

**Purpose:**

Attention-deficit/hyperactivity disorder (ADHD) is a neurodevelopmental condition characterized by inattention, hyperactivity, and impulsivity. A positive association between ADHD and obesity has been observed, especially in adult samples. In this study, prevalence and correlates of self-reported symptoms indicative of a positive screening for ADHD were examined in patients seeking bariatric treatment.

**Material and Methods:**

The study sample was composed of 260 adult patients with obesity referred for bariatric surgery to the Obesity Center of the Endocrinology Unit in Pisa University Hospital between January 2006 and November 2016 (BMI ≥ 30 kg/m^2^; mean ± standard deviation = 46.27 ± 7.45 kg/m^2^). ADHD symptoms were identified using ADHD Symptom Check‐List‐90‐R Screening Scale. Night-eating, binge-eating/purging behaviors, and temperamental and character traits were assessed in a subsample of 95 patients.

**Results:**

Thirty participants had a positive screening for ADHD (11.5%, 95% CI = 7.9–16.1%). Patients with a positive screening showed significantly higher rates of anxiety disorders (40% vs. 16.5%, *χ*^2^ = 7.97, *p* = 0.005) panic disorder (40% vs. 14.3%, *χ*^2^ = 10.48, *p* = 0.001), and a higher severity of psychopathological symptoms and sleep disturbances than those without. In subsample analyses, ADHD symptoms severity was associated with more bulimic behaviors (*r* = 0.31–0.46), greater harm avoidance (*r* = 0.45–0.66), less self-directedness (*r* =  − 0.44–0.63), and cooperativeness (*r* =  − 0.26–0.42).

**Conclusion:**

ADHD symptoms may be common in patients with obesity seeking bariatric treatment and are positively associated with disordered eating, internalizing features, and maladaptive character traits.

*Level of Evidence*: V, cross sectional descriptive study.

**Graphical Abstract:**

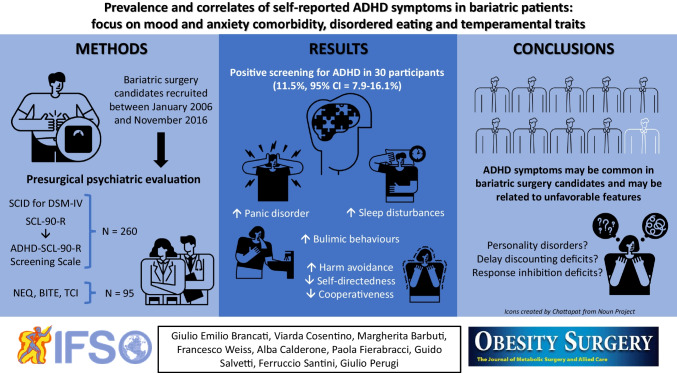

## Introduction

Attention-deficit/hyperactivity disorder (ADHD) is a neurodevelopmental condition characterized by inappropriate levels of inattention, hyperactivity, and impulsivity [1–6]. High rates of comorbidity can be found between ADHD and a variety of psychiatric disorders, especially mood, anxiety, substance use, conduct, and personality disorders [7–11]. Youths with ADHD present a significantly increased risk of developing later substance abuse or dependence [12, 13], major depression [14, 15], and bipolar disorder [16]. ADHD is also associated with specific medical conditions, with a growing interest for the connection between ADHD and obesity. Both are chronic conditions that represent a relevant public and clinical health concern globally [17].

The prevalence of ADHD in clinical samples of patients in care for obesity is higher than in the general population [18], with up to over one-fourth of patients with obesity being diagnosed with predominantly inattentive ADHD [19]. This association is more pronounced in adolescents and adults than in children [20], and it is crucial for gaining a better understanding of the development of severe obesity [21].

Many pathogenetic mechanisms and dysfunctional pathways have been proposed to underlie the association between obesity and ADHD [18, 22]. First, it has been argued that ADHD and obesity share common genetics and neurobiological mechanisms that involve the dopaminergic system and brain reward pathways. Functional magnetic resonance imaging studies have identified a significant neural overlap in circuits associated with reward, response inhibition, and emotional processing and regulation in individuals with ADHD, obesity, and abnormal eating behavior [23]. Recent genetic and prenatal studies have further supported the bidirectional association between these conditions, encompassing both genetic and early environmental origins [22, 24].

From a clinical standpoint, obesity can also be considered, in comorbid cases, secondary to deficits in inhibitory control and executive functions associated with ADHD. Executive dysfunctions have been observed in patients with obesity [25] and may lead to disordered eating behaviors and feeding anomalies, such as binge-eating or bulimic behaviors, skipping meals, emotional hunger, eating at inappropriate times, and night-eating [22, 26]. Anomalies of the dopamine system may also lead to decreased physical activity and to a more sedentary lifestyle, combined with the consumption of highly palatable or caloric food, that activate the dopamine-reward pathways and could be used as self‐medication [27]. In addition, sleep problems encountered by individuals with ADHD, including disruptions in circadian rhythm and shortened sleep duration resulting from delayed onset of melatonin, have already been associated with the development of obesity [28, 29].

ADHD could potentially contribute to treatment challenges in patients with obesity participating in weight loss programs [30]. Although findings on the effect of ADHD on BMI change in patients undergoing bariatric surgery were mixed, ADHD symptoms have been associated with lower adherence to scheduled follow-up visits after the intervention [31], and there is some evidence that ADHD treatment might potentially improve the outcomes of weight-loss strategies in comorbid individuals [32]. From a different perspective, impulsivity, a core symptom domain of ADHD, has been proposed as a transdiagnostic psychological construct contributing to eating behavior and obesity, as well as to suboptimal outcomes of bariatric surgery [3]. Impulsivity refers both to the inability to inhibit automatic behavior in response to salient stimuli (i.e., response inhibition) and to the tendency to discount future consequences in favor of immediate rewards (i.e., delay discounting) [3]. Deficits of response inhibition and delay discounting, which are central to neuropsychological theories of ADHD [33, 34], have been observed in patients with obesity [35–37] and predict weight loss through obesity intervention [38–40]. Given the evidence of positive effects of ADHD treatment on response inhibition and delay discounting [41–43], diagnosing and treating ADHD in patients with obesity might help improve eating behavior and bariatric surgery outcomes.

Despite the potential significance of the comorbidity between these conditions, only a limited number of studies have explored the prevalence of ADHD or ADHD symptoms among patients with obesity seeking bariatric surgery [44–51]. Importantly, one of the most commonly used instruments to assess psychopathology in candidates for bariatric surgery, the Structured Clinical Interview for DSM-IV Axis I Disorders – Clinical Version (SCID-I) [52], does not include any section on adult ADHD [52]. Accordingly, ADHD has not been considered in several studies on the prevalence of psychiatric disorders in bariatric samples [53–58].

Even fewer studies focused on the psychopathological correlates of ADHD symptoms in these patients [45, 48]. Particularly, Gruss et al. investigated differences in demographic variables, history of psychotherapeutic contact, depressive symptoms and disorders, screening for binge-eating disorder, and daytime sleepiness between patients screening positive or negative for adult ADHD [48], while Alfonsson et al. examined the correlations among ADHD, anxious, depressive symptoms, and food cravings [45]. None of these studies focused on the associations of ADHD symptoms with other formally diagnosed affective disorders, such as bipolar disorder, anxiety disorders, and eating disorders. Additionally, they did not explore connections with other psychopathological dimensions, including bulimic and night-eating behaviors, as well as temperamental traits.

For a better identification of patients potentially impacted by ADHD within patients with obesity, an exploration of ADHD-related dimensions becomes crucial. In this post hoc analysis of data from a previously described sample of patients with obesity referred for bariatric surgical treatment [59], we examined the prevalence and the psychiatric comorbidity with mood, anxiety, and eating disorders of self-reported symptoms indicative of a positive screening for ADHD [3]. Differences in eating behavior and temperamental traits between participants with obesity with and without a positive screening for ADHD were also assessed.

Based on previous studies, significant positive associations between ADHD symptoms and bipolar or related disorders, sleep disturbances, and bulimic behaviors are expected. Importantly, this was the first study to investigate on temperament and ADHD symptoms in patients with obesity. Based on the available data in adult samples without obesity [60, 61], higher novelty seeking and harm avoidance, and lower self-directedness are expected to be associated with ADHD symptoms.

## Patients and Methods

### Recruitment and Assessment

The study sample was composed of 260 patients with obesity (BMI ≥ 30 kg/m^2^) referred for evaluation for bariatric surgery to the Obesity Center of the Endocrinology Unit in Pisa University Hospital between January 2006 and November 2016.

All patients were adult (≥ 18 years) and provided written informed consent to data collection for research purposes. Partial overlapping samples and procedures were previously described in studies from our group [5, 59]. Particularly, clinical charts of patients were retrieved and examined. For any given patient, two independent researchers retrieved the data and filled a pre-defined data abstraction form. Any disagreement was resolved by consensus after discussion.

All the medical records available were searched for sociodemographic information, anthropometric variables, i.e., weight and BMI, and lifetime diagnoses of mood, anxiety, and eating disorders. During the presurgical evaluation, all patients underwent standard interviews conducted by experienced licensed psychiatrists. In a single session, both current and lifetime psychiatric diagnoses were assessed. The SCID-I Clinical Version [52] was utilized to systematically assess symptoms of major depressive disorder, bipolar spectrum disorders (including BD type 1 and 2, and other specified bipolar disorder), anxiety disorders, and eating disorders (specifically anorexia and bulimia nervosa). Since the SCID-I was routinely used as part of clinical presurgical evaluations, no specific training was provided for the purposes of this study. The psychiatrists who conducted the interviews had received training during their education and had extensive experience in administering the SCID-I. Due to the nature of the interviews, a thorough assessment of the history of anorexia, bulimia, and binge-eating disorder was further conducted in accordance with DSM-IV-TR criteria [62]. Lifetime diagnoses of mood, anxiety, and eating disorders finally reported by clinicians according to DSM-IV-TR criteria after comprehensive evaluation of all the available information (e.g., information from other informants, review of past records, family history, treatment history) were recorded for this study.

The Symptom Checklist-90-Revised (SCL-90-R) was routinely used to investigate the severity of current general psychopathological symptoms and distress [63]. The SCL-90-R is a multidimensional self-rated measure consisting of 90 items rated on a five-point scale from 0 (“Not at All”) to 4 (“Extremely”) specifying how much each symptom has bothered during the past 7 days. Items are assembled into nine symptom dimensions, namely somatization, obsessive–compulsive, interpersonal sensitivity, depression, anxiety, hostility, phobic anxiety, paranoid ideation, and psychoticism. Three additional items investigate sleep disturbances. In bariatric patients, good internal consistency of all subscales (Cronbach’s *α* = 0.77–0.90) and convergent validity with the data gathered in the clinical interviews have been demonstrated [64]. A recent study also found good discriminant validity of the Italian version of the scale for binge-eating disorder and major depression in bariatric surgery candidates (area under the curve ≥ 0.70) [65]. Only patients having completed the SCL-90-R during pre-surgical consultations were included in this study.

ADHD symptoms were identified using the ADHD‐SCL‐90‐R screening scale [66], a recently developed screening instrument composed of 16 items selected from SCL-90-R, based on content match with items from the Conners’ Adult ADHD rating scale [67], an established questionnaire measuring adult ADHD symptoms. The items, originally belonging to the obsessive–compulsive, interpersonal sensitivity, depression, anxiety, and hostility dimensions of the SCL-90-R, are grouped in four subscales, which respectively capture Problems with Self‐Concept, Impulsivity/Emotional Lability, Inattention/Memory Problems, and Hyperactivity/Restlessness. As for the SCL-90-R, items are rated on a five-point Likert scale from 0 to 4. High significant correlations (*r* = 0.57–0.59) with ADHD‐specific scales have been evidenced. Compared to the other established ADHD‐specific tools, the ADHD‐SCL‐90‐R screening scale showed comparably good psychometric properties, except for a lower specificity. Overall, good to excellent internal consistency (Cronbach’s *α* = 0.79–0.90) and an acceptable discriminatory power (area under the curve = 0.74) was found in a sample of 412 individuals examined by an ADHD Special Consultations Unit [66]. The cut‐off value of 19 (i.e., total score ≥ 19) was chosen to detect patients with a positive screening for ADHD in our sample. This threshold was previously suggested by Abbass and colleagues, who reported a sensitivity of 78% and a specificity of 56% when utilizing that specific cut-off [66].

Disordered eating behaviors and temperamental traits were further investigated in a subsample of 95 patients (36.5% of the whole sample) who completed the Night Eating Questionnaire (NEQ) [68, 69], the Bulimic Investigatory Test, Edinburgh (BITE) [70, 71], and the Temperament and Character Inventory (TCI) [72, 73].

The NEQ, a commonly used screening instrument for night-eating disorder, consists of 14 self-rated items subdivided into four facets: morning anorexia, evening hyperphagia, mood/sleep, and nocturnal ingestions [68, 69]. Adequate internal consistency (Cronbach’s *α* = 0.70), convergent validity with measures of night eating, disordered eating, sleep, mood, and stress, and appropriate discriminant validity for night-eating syndrome (positive predictive value = 72.7%) were found in bariatric surgery candidates [68]. Similar internal consistency (Cronbach’s *α* = 0.48–0.71), positive correlations with measures of disordered eating, sleep, and mood, and adequate test–retest reliability (intraclass correlation coefficient = 0.68) were observed for the Italian version of the questionnaire [69].

The BITE is a self-report measure composed of two subscales: the symptom scale (30 items in yes/no format), which assess disordered eating and related psychological symptoms, and the severity scale (three items on a Likert scale), which evaluates the frequency of fasting, purging, and binge-eating behaviors [70, 71]. Good reliability (Cronbach’s *α* = 0.62–0.96; test–retest *r* = 0.68–0.86), excellent discriminant validity, convergence with other measures of disordered eating, and sensitivity to change were demonstrated in samples including patients with binge-eating behaviors and healthy controls [71]. The Italian version of the test has proved high internal consistency (Cronbach’s *α* = 0.82–0.91) and good discriminant validity for binge-eating disorder in women with obesity [70].

Finally, the TCI was used to evaluate temperamental traits based on Cloninger’s psychobiological theory of temperaments [72, 73]. It consists of 240 items, answered yes or no, exploring four temperamental dimensions (i.e., novelty seeking, harm avoidance, reward dependence, and persistence) and three character facets (i.e., self-directedness, cooperativeness, and self-transcendence). Adequate internal consistency and test–retest reliability have been repeatedly reported for TCI scales both in clinical [73, 74] and nonclinical samples [72, 75, 76].

### Statistical Analyses

All the statistical analyses were performed using R Statistical Software (Foundation for Statistical Computing, Vienna, Austria) between January and June 2022. Shapiro–Wilk test was used to exclude normality. First, patients with and without a positive screening for ADHD were compared using Pearson’s chi-squared tests (or Fisher’s exact test, when needed) for gender and psychiatric comorbidity, and Wilcoxon rank-sum test for age, BMI and SCL-90-R subscales that did not included items overlapping with ADHD‐SCL‐90‐R screening scale (i.e., somatization, phobic anxiety, paranoid ideation, psychoticism, sleep) (*N* = 260).

Eating pattern and temperamental differences between patients with and without a positive screening were the evaluated in the subsample (*N* = 95) using Wilcoxon rank-sum test (or Student’s *t* test when comparing TCI-NS scores). Finally, the associations between ADHD symptoms severity as measured by ADHD‐SCL‐90‐R screening scale and its subscales and scales assessing eating behaviors and temperamental traits were tested using Spearman’s rank correlation coefficient (*N* = 95). This latter approach allowed to examine whether differential correlates of separate ADHD dimensions could be highlighted.

Non-parametric tests were used for continuous variables after exclusion of normality using Shapiro–Wilk test. A statistical significance level of *p* < 0.05 was set for all tests, after false discovery rate (FDR) correction for multiple comparisons.

## Results

The sample was composed of 260 patients with obesity referred for bariatric surgery. Most patients were female (*N* = 187, 71.9%). Age ranged between 18 and 66 years, with a mean of 44.31 ± 10.71 years. Weight ranged between a minimum of 81.8 kg to a maximum of 221 kg, with a mean of 127.88 ± 24.53 kg. BMI ranged between 31.95 and 76.95 kg/m^2^, with a mean of 46.27 ± 7.45 kg/m^2^. Most patients were diagnosed with class II obesity (35 kg/m^2^ ≤ BMI < 40 kg/m^2^), with 208 out of 260 patients affected (80%). The other patients were diagnosed with class III obesity (BMI ≥ 40 kg/m^2^; *N* = 47, 18.1%), except for five (1.9%) who were affected by class I obesity (BMI < 35 kg/m^2^) and were referred for presurgical evaluation due to severe obesity complications or comorbidities. Similar characteristics were evidenced in patients’ subsample (Table 1).

**Table 1:** Demographic and clinical characteristics of the samples

	Whole sample (*N* = 260)	Subsample (*N* = 95)
	Mean ± SD / N (%)	Mean ± SD/*N* (%)
Demographic and anthropometric variables		
Age (years)	44.31 ± 10.71	43.43 ± 10.47
Gender (female)	187 (71.9%)	72 (75.8%)
BMI (kg/m^2^)	46.27 ± 7.45	46.69 ± 6.8
Lifetime psychiatric comorbidity and family history		
Any mood disorder	97 (37.3%)	44 (46.3%)
Major depressive disorder	40 (15.4%)	13 (13.7%)
Bipolar or related disorder	57 (21.9%)	31 (32.6%)
Bipolar disorder type 1	5 (1.9%)	3 (3.2%)
Bipolar disorder type 2	33 (12.7%)	17 (17.9%)
Cyclothymia or other specified BDs	19 (7.3%)	11 (11.6%)
Family history of mood disorders	44 (16.9%)	21 (22.1%)
Any anxiety disorder	50 (19.2%)	19 (20%)
Panic disorder	45 (17.3%)	19 (20%)
Family history of anxiety disorders	18 (6.9%)	7 (7.4%)
Any eating disorders	91 (35%)	34 (35.8%)
Binge-eating disorder	89 (34.2%)	33 (34.7%)
Bulimia nervosa	7 (2.7%)	4 (4.2%)

Notably, according to some guidelines [77], bariatric surgery could be considered for patients with BMI ≥ 35–40 kg/m^2^ with associated comorbidities that are expected to improve with weight loss, as well as for patients with BMI ≥ 30–35 kg/m^2^ and type 2 diabetes and/or hypertension with poor control despite optimal medical therapy. Not all patients included were considered eligible for bariatric surgery after the evaluation.

Thirty participants were identified as having a positive screening for ADHD according to ADHD‐SCL‐90‐R screening scale (11.5%, 95% CI = 7.9–16.1%). A similar prevalence was observed in patients’ subsample (13 of 95, 13.7%, 95% CI = 7.5–22.3%).

Differences in demographic and anthropometric characteristics, lifetime psychiatric comorbidity, and SCL-90-R subscales not including items overlapping with ADHD‐SCL‐90‐R screening scale were assessed in the whole sample (*N* = 260) (Table 2). No significant differences emerged for age, gender, and BMI. Patients with a positive screening for ADHD were significantly more frequently diagnosed with anxiety disorders in comparison with patients without. Panic disorder was specifically significantly associated with a positive screening for ADHD. Mood disorders, particularly bipolar disorder type 2 and bipolar or related disorders in general, were more frequently diagnosed in patients with a positive screening for ADHD than in those without; however, these differences did not survive FDR correction for multiple comparisons. No significant differences were observed for eating disorders and family history of mood and anxiety disorders. Significant differences in all the non-overlapping SCL-90-R subscales were observed: patients with a positive screening for ADHD showed significantly higher scores on somatization, phobic anxiety, paranoid ideation, psychoticism, and sleep disturbances compared to unaffected participants.

**Table 2:** Differences between patients with and without positive screening for attention-deficit/hyperactivity disorder symptoms (ADHD). Effect size (ES) measures include Wilcoxon’s *r* for comparisons of continuous variables, mean square contingency coefficient (*φ*) for categorical variables. *χ*^2^ are reported for chi-squared tests. False discovery rate (fdr) correction for multiple comparisons was applied. *p* < 0.05 are shown in bold

	Positive screening for ADHD (*N* = 30)	Negative screening for ADHD (*N* = 230)				
	Mean ± SD/*N* (%)	Mean ± SD *N* (%)	*χ*^2^	ES	*p*	*p*_fdr_
Demographic and anthropometric data						
Age (years)	45.53 ± 9.88	44.15 ± 10.83	-	0.04	0.533	NS
Gender (female)	23 (76.7%)	164 (71.3%)	0.16	0.04	0.690	NS
BMI (kg/m^2^)	47.49 ± 8.02	46.11 ± 7.38	-	0.05	0.449	NS
Lifetime psychiatric comorbidity and familiarity						
Any mood disorder	17 (56.7%)	80 (34.8%)	4.54	0.15	0.033	NS
Major depressive disorder	5 (16.7%)	35 (15.2%)	-	0.01	0.791	NS
Bipolar or related disorders	12 (40%)	45 (19.6%)	5.34	0.16	**0.021**	NS
Bipolar disorder type 1	2 (6.7%)	3 (1.3%)	-	0.13	0.103	NS
Bipolar disorder type 2	8 (26.7%)	25 (10.9%)	-	0.15	**0.035**	NS
Cyclothymia or other specified BDs	2 (6.7%)	17 (7.4%)	-	-0.01	1.000	NS
Family history of mood disorders	7 (23.3%)	37 (16.1%)	0.54	0.06	0.461	NS
Any anxiety disorder	12 (40%)	38 (16.5%)	7.97	0.19	**0.005**	** < 0.05**
Panic disorder	12 (40%)	33 (14.3%)	10.48	0.22	**0.001**	** < 0.05**
Family history of anxiety disorders	4 (13.3%)	14 (6.1%)	-	0.09	0.139	NS
Any eating disorders	13 (43.3%)	78 (33.9%)	0.66	0.06	0.416	NS
Binge-eating disorder	13 (43.3%)	76 (33%)	0.83	0.07	0.361	NS
Bulimia nervosa	1 (3.3%)	6 (2.6%)	-	0.01	0.581	NS
Symptom Checklist-90-Revised						
Somatization	1.71 ± 0.63	0.64 ± 0.47	-	0.47	** < 0.001**	** < 0.001**
Phobic anxiety	0.65 ± 0.65	0.11 ± 0.23	-	0.41	** < 0.001**	** < 0.001**
Paranoid ideation	1.55 ± 0.77	0.41 ± 0.44	-	0.46	** < 0.001**	** < 0.001**
Psychoticism	0.95 ± 0.66	0.17 ± 0.22	-	0.45	** < 0.001**	** < 0.001**
Sleep	1.6 ± 1.24	0.63 ± 0.75	-	0.27	** < 0.001**	** < 0.001**

Differences in NEQ, BITE and TCI subscales were evaluated within the study subsample (*N* = 95) (Table 3). Patients with a positive screening for ADHD scored significantly higher on NEQ mood/sleep subscale, BITE symptom scale, and TCI harm avoidance scale compared to patients without. Significantly lower scores on the TCI self-directedness and cooperativeness scales were found in patients with a positive screening for ADHD. Higher NEQ total scores were also observed in patients with a positive screening; however, the difference was not significant after FDR correction.

**Table 3:** Eating pattern and temperamental differences between patients with and without positive screening for attention-deficit/hyperactivity disorder symptoms (ADHD). Effect size (ES) measures include Wilcoxon’s *r* or Cohen’s *d* (*§*). Student’s *t* is reported for Student’s *t*-test. False discovery rate (fdr) correction for multiple comparisons was applied. *p* < 0.05 are shown in bold

	Positive screening for ADHD (*N* = 13)	Negative screening for ADHD (*N* = 82)				
	Mean ± SD	Mean ± SD	*t*	ES	*p*	*p*_fdr_
Night Eating Questionnaire						
Total score	14 ± 7.99	9.54 ± 4		0.22	**0.029**	NS
Morning anorexia	1.54 ± 0.78	1.67 ± 0.89		− 0.06	0.590	NS
Evening hyperphagia	3.77 ± 1.79	3.63 ± 1.48		0.07	0.530	NS
Mood/sleep	5.23 ± 2.65	2.76 ± 2.07		0.32	**0.002**	** < 0.01**
Nocturnal ingestions	2 ± 5.29	0.67 ± 2.11		0.07	0.477	NS
Bulimic Investigatory Test, Edinburgh						
Symptom scale	20.38 ± 9.16	11.94 ± 7.39		0.32	**0.002**	** < 0.01**
Severity scale	1.46 ± 1.2	1.54 ± 1.89		0.06	0.575	NS
Temperament and Character Inventory						
Novelty seeking^§^	17.85 ± 6.04	18.27 ± 4.29	− 0.24	− 0.09	0.812	NS
Harm avoidance	22.31 ± 5.66	15.87 ± 5.73		0.33	**0.001**	** < 0.01**
Reward dependence	14.54 ± 3.53	15.3 ± 3.12		− 0.06	0.537	NS
Persistence	4.38 ± 1.33	4.46 ± 1.6		− 0.02	0.830	NS
Self-directedness	23.38 ± 5.66	32.37 ± 7.02		− 0.40	** < 0.001**	** < 0.005**
Cooperativeness	29.15 ± 6.57	33.6 ± 4.67		− 0.25	**0.014**	** < 0.05**
Self-transcendence	12.85 ± 6.77	10.33 ± 5.29		0.12	0.235	NS

The associations between NEQ, BITE, and TCI subscales and ADHD‐SCL‐90‐R screening scale total and subscales scores were also tested according to a dimensional approach, to evaluate whether different ADHD facets could be specifically associated with disordered eating behavior and/or with distinct temperament and character dimensions. A similar pattern of correlations was observed for ADHD‐SCL‐90‐R screening scale total score and for each of its subscales, corresponding to previously observed differences between patients with and without a positive screening for ADHD (Table 4).

**Table 4:** Associations between ADHD symptoms, eating patterns and temperamental traits (*N* = 95). Spearman’s *r* are reported. Uncorrected *p*-values are shown. All significant correlations that stayed significant after false discovery rate correction for multiple comparisons (*p*_fdr_ < 0.05) are shown in bold

	ADHD‐SCL‐90‐R		ADHD‐SCL‐90‐R‐PSC		ADHD‐SCL‐90‐R‐IM/EL		ADHD‐SCL‐90‐R‐IA/MP		ADHD‐SCL‐90‐R‐HY/RL	
	*r*	*p*	*r*	*p*	*r*	*p*	*r*	*p*	*r*	*p*
Night Eating Questionnaire										
Total score	0.40	** < 0.001**	0.25	**0.016**	0.32	**0.001**	0.35	**0.001**	0.34	**0.001**
Morning anorexia	0.05	0.620	− 0.04	0.665	0.11	0.311	0.12	0.237	0.03	0.759
Evening hyperphagia	0.03	0.764	0.02	0.825	0.12	0.265	− 0.04	0.689	0.02	0.876
Mood/sleep	0.50	** < 0.001**	0.44	** < 0.001**	0.36	** < 0.001**	0.40	** < 0.001**	0.42	** < 0.001**
Nocturnal ingestions	0.15	0.158	0.08	0.468	0.01	0.913	0.16	0.113	0.13	0.193
Bulimic Investigatory Test, Edinburgh										
Symptom score	0.44	** < 0.001**	0.43	** < 0.001**	0.31	**0.003**	0.46	** < 0.001**	0.31	**0.002**
Severity score	0.12	0.237	0.16	0.112	0.06	0.559	0.06	0.548	0.14	0.190
Temperament and Character Inventory										
Novelty seeking	0.06	0.562	0.01	0.895	0.03	0.763	0.12	0.254	− 0.04	0.680
Harm avoidance	0.66	** < 0.001**	0.67	** < 0.001**	0.45	** < 0.001**	0.55	** < 0.001**	0.59	** < 0.001**
Reward dependence	0.00	0.986	− 0.16	0.133	− 0.03	0.799	0.04	0.727	0.07	0.485
Persistence	− 0.02	0.822	− 0.12	0.257	0.05	0.654	− 0.11	0.275	0.09	0.381
Self-directedness	− 0.63	** < 0.001**	− 0.63	** < 0.001**	− 0.52	** < 0.001**	− 0.58	** < 0.001**	− 0.44	** < 0.001**
Cooperativeness	− 0.38	** < 0.001**	− 0.42	** < 0.001**	− 0.29	**0.004**	− 0.32	**0.002**	− 0.26	**0.011**
Self-transcendence	0.20	0.056	0.21	0.037	0.21	0.042	0.12	0.265	0.15	0.147

Subscales of the ADHD‐SCL‐90‐R screening scale: ADHD‐SCL‐90‐R‐PSC = Problems with Self‐Concept; ADHD‐SCL‐90‐R‐IM/EL = Impulsivity/Emotional Lability; ADHD‐SCL‐90‐R‐IA/MP = Inattention/Memory Problems; ADHD‐SCL‐90‐R‐HY/RL = Hyperactivity/Restlessness

## Discussion

A positive screening for ADHD was found in 11.5% of our sample (*N* = 260) and was significantly associated with anxiety disorders, especially panic disorder. Patients who had a positive screening showed a significantly higher severity of psychopathological symptoms and sleep disturbances compared to those unaffected. In contrast with our hypothesis, mood disorders, particularly bipolar disorder type 2 and bipolar or related disorders in general, were associated with a positive screening for ADHD only at the uncorrected level of analysis. No significant association with eating disorders, nor with gender, was observed. However, in subsample analyses (*N* = 95), ADHD symptoms were associated with mood and sleep problems, bulimic behaviors, greater harm avoidance, and lower self-directedness and cooperativeness.

The proportion of patients screening positive for adult ADHD was two to four times higher than the prevalence of adult ADHD in the general population [78–80]. This rate was consistent with the 12% prevalence of ADHD reported in youths with obesity aged 10 to 17 years [81]. Higher rates of ADHD or ADHD symptoms were previously repeatedly reported in adult samples with obesity, with estimates ranging between 27 and 38% in multiple studies [19, 46, 47, 51, 82]. Nevertheless, most of the studies had low sample sizes and only few reports included more than one hundred of patients [19, 45, 48, 50, 51]. Among these latter, prevalence rates ranged from 5.6% [50] to 27.4–28.3% [19, 51] in studies based on clinical interviews, and hovered around 10.2–12.1% in studies relying on self-report screening measures [45, 48]. A similar proportion of patients screening positive for adult ADHD (8.9%) was found in 90 individuals with severe obesity considering bariatric surgery [49].

Our finding is consistent with estimates from previous studies using self-report screening questionnaires in patients referred to bariatric surgery. The discrepancy with other studies may be attributed to differences in setting and assessment methods. First, it could be hypothesized that less patients with ADHD or with ADHD symptoms, such as disorganization or impulsivity, are referred to bariatric surgery than to other weight loss programs. Moreover, it could be possible that screening measures developed in the general population may have less validity in patients with obesity, especially during presurgical evaluations. However, it could not be excluded that the higher prevalence obtained through patients’ interviews in other studies may have been partly inflated by lack of third-party information on childhood behavior.

As concerns psychiatric comorbidity, in our study, the strongest association of a positive screening for ADHD was observed for anxiety disorders, especially panic disorder. Comorbidity between panic disorder and ADHD has been seldom examined and mixed results emerged from comparisons between adults with ADHD and healthy controls [83, 84]. Although panic disorder was found to be the least common anxiety disorder in children with ADHD [85] and, in a Japanese study, the polygenic risk for ADHD was found to be negatively associated with panic disorder [86], a recent study found a relatively high comorbidity with panic disorder in adults with ADHD, second only to social anxiety disorder [87]. Importantly, stronger correlations between ADHD and anxiety symptoms than between ADHD and depressive symptoms have been previously observed in bariatric surgery candidates [45]. It may be posited that demographic factors, such as female gender predominance [84], and specific ADHD features, such as predominantly inattentive presentation and sluggish cognitive tempo, previously associated both with obesity [19, 88] and anxiety comorbidity [89], may be related to the high prevalence of anxiety disorders in patients with obesity reporting ADHD symptoms. Interestingly, lifetime anxiety disorders have been recently associated with greater impairments in delay discounting in patients seeking bariatric surgery [50].

Eating disorders were not significantly overrepresented among our patients with obesity with a positive screening for ADHD. However, when evaluating the eating pattern using the BITE questionnaire, patients who screened positive for ADHD scored significantly higher than those who did not on the symptom scale, but not on the severity scale. This implies a presence of more disordered eating behaviors, though not a higher frequency of bulimic symptoms. While population studies generally supported the association between ADHD and eating disorders [8], previous studies conducted in samples with obesity failed to identify increased rates of binge-eating disorder diagnoses in patients with ADHD [48, 49]. However, ADHD symptoms were positively associated with disordered eating patterns, loss of control over eating, and emotional cravings in candidates for bariatric surgery [45].

Eating behaviors not specifically addressed by current nosographic conceptualizations, such as loss of control over eating, craving for palatable foods, and emotional hunger, may be especially relevant in patients with obesity with ADHD or significant ADHD symptoms and warrant further investigation [8, 27, 90, 91]. Among these constructs, night-eating was specifically investigated in our study. While sleep disturbances were more frequently reported by patients with a positive screening for ADHD, no significant associations with nocturnal ingestions, nor with evening hyperphagia were observed. In contrast, a previous study found that, among patients with obesity, those screening positive for ADHD reported more frequently than the others to wake up at night to eat [91]. Moreover, according to an online survey of university students, those diagnosed with night-eating syndrome had more often than others a history of ADHD [92]. Given the paucity of studies, both in patients with and without obesity, more research is needed.

Finally, temperament and character dimensions were evaluated. ADHD symptoms were positively associated with harm-avoidant temperamental traits and negatively associated with self-directedness and cooperativeness character facets. Both high harm avoidance, the tendency to inhibit or avoid responses to aversive stimuli, and low self-directedness, the capacity to control, regulate, and modify behavior to fulfil one’s objectives and uphold one’s values, had been previously strongly associated with ADHD in adult samples [61]. Low self-directedness may reflect deficits in executive functioning and self-regulation observed in patients with ADHD [93], and may be associated with suboptimal outcomes of obesity treatment [94]. Also, cooperativeness, the propensity for identification with, and acceptance of others, has shown moderate negative associations with ADHD in previous studies [61].

Surprisingly, instead, no significant association was observed with novelty seeking, the tendency to approach novel situations for rewards. Novelty seeking had been previously strongly associated with ADHD both in adults and in children [61]. However, while novelty-seeking shows a closer relationship with hyperactivity/impulsivity symptoms, higher inattention symptoms have been associated with decreased self-directedness and increased harm avoidance [95, 96]. Predominantly inattentive manifestations of ADHD in patients with obesity could thereby explain the lack of association between ADHD symptoms and novelty-seeking in our sample.

Interestingly, a cluster analysis applied to TCI scores of 463 patients with obesity and binge-eating behaviors revealed a cluster of patients with higher harm avoidance and lower self-directedness showing greater depressive symptoms, higher eating impulsivity, more problems with body image and poorer quality of life, but no differences in BMI or prevalence of binge-eating disorder vs. eating disorder not otherwise specified [97], whether ADHD might subtend the manifestations of this more complex variant of eating disorders in patients with obesity warrant more investigation in future studies.

Importantly, high harm avoidance, low self-directedness, and low cooperativeness have been found to characterize, among women with ADHD, those with more pronounced borderline personality traits [98]. Personality disorders have been associated with suboptimal clinical outcomes of bariatric surgery [99]. Considering the potential benefits of treating ADHD in patients with personality disorders [100, 101], the assessment of ADHD could merit further attention in patients with personality disorders seeking bariatric treatment.

Some important limitations of this study should be considered. First, the cross-sectional study design limited the assessment of psychiatric comorbidity to retrospective accounts, which may be at risk of recall bias. Moreover, information on remission status or current psychopharmacotherapy were not systematically recorded. More importantly, ADHD was not systematically investigated by the evaluating clinician, and no retrospective assessment of childhood symptoms was conducted. The recognition of ADHD symptoms was based on a self-report screening questionnaire rather than on a structured diagnostic interview performed by an experienced psychiatrist, which limits the validity of the results. Indeed, while the validation of the screening tool used was performed in an outpatient ADHD consultation unit, where the proportion of individuals with ADHD was higher than in the general population, no studies were conducted in community samples, where lower positive predictive values are expected based on the prevalence of ADHD. In addition, the recruitment occurred during routinely performed presurgical evaluations, which could lead some patients to underreport psychopathological symptoms to avoid to prejudice surgery.

Despite these flaws, our findings are in line with previous studies reporting similar prevalence of patients screening positive for ADHD in bariatric samples. While preliminary, our study benefited from a relatively large sample size and contributed to characterizing a potential psychological and behavioral phenotype in patients with obesity marked by ADHD symptoms, anxiety disorders, bulimic behaviors, polymorphic psychopathological manifestations, sleep disturbances, and maladaptive character traits. Drawing from previous studies, it might be hypothesized that these patients could also exhibit a higher severity of depressive symptoms [48, 97], more psychotherapy contact in the past [48], poorer body image and quality of life [97], higher eating impulsivity and food cravings [45, 97], and potentially poorer outcomes of obesity treatment [30–32, 94].

It remains to be explored whether a categorical diagnosis of ADHD or, alternatively, transdiagnostic psychopathological features, such as impulsivity or emotional dysregulation, or neuropsychological impairments of executive functioning better capture the complexity of patients with obesity and more severe psychopathology, as well as predict therapeutic outcomes. Further studies assessing both adulthood and childhood ADHD symptoms through validated clinical interviews as well as related attention deficits and executive dysfunctions through neuropsychological testing needs to be done to establish the prevalence of ADHD and to confirm or confute our findings and hypotheses. Longitudinal studies are also necessary to assess whether the phenotype outlined in this study, and which specific features within it, might be predictive of unfavorable surgery outcomes in bariatric patients. Moreover, a larger range of overweight conditions, as well as a controlled design, will be needed in future studies to investigate whether different ADHD features are more prevalent in patients with different BMI.

From a clinical perspective, diagnosing and treating ADHD in patients with obesity may have relevant implications. As previously mentioned, there is some indication that ADHD treatment might potentially reduce psychopathology severity in patients with comorbid personality disorders [100, 101] and improve response inhibition and delay discounting [41–43]. These changes, albeit indirectly, could potentially have a positive impact on bariatric surgery outcomes. Additionally, ADHD treatment might support long-term weight loss in individuals with suboptimal clinical response [32].

Based on our findings, patients with obesity showing comorbid anxiety disorders, especially panic disorder, high severity of psychopathological symptoms, sleep disturbances, bulimic behaviors, and specific character traits may screen positive for ADHD and, consequently, deserve further clinical assessment and management before bariatric surgery.

## Data Availability

The datasets generated during and/or analyzed during the current study are available from the corresponding author on reasonable request.
